# Analysis of PSPHL as a Candidate Gene Influencing the Racial Disparity in Endometrial Cancer

**DOI:** 10.3389/fonc.2012.00065

**Published:** 2012-07-04

**Authors:** Jay E. Allard, Gadisetti V. R. Chandramouli, Katherine Stagliano, Brian L. Hood, Tracy Litzi, Yutaka Shoji, Jeff Boyd, Andrew Berchuck, Thomas P. Conrads, G. Larry Maxwell, John I. Risinger

**Affiliations:** ^1^Walter Reed Army Medical CenterWashington, DC, USA; ^2^Department of Obstetrics, Gynecology and Reproductive Biology, Michigan State University College of Human MedicineGrand Rapids, MI, USA; ^3^Curtis and Elizabeth Anderson Cancer Institute at Memorial Health University Medical CenterSavannah, GA, USA; ^4^Women’s Health Integrated Research Center at Inova Health SystemAnnandale, VA, USA; ^5^Fox Chase Cancer CenterPhiladelphia, PA, USA; ^6^Division of Gynecologic Oncology, Duke UniversityDurham, NC, USA

**Keywords:** endometrial cancer, PSPHL, racial disparity

## Abstract

Endometrial cancer is the most commonly diagnosed gynecologic malignancy in the United States. A well recognized disparity by race in both incidence and survival outcome exists for this cancer. Specifically Caucasians are about two times more likely to develop endometrial cancer than are African-Americans. However, African-American women are more likely to die from this disease than are Caucasians. The basis for this disparity remains unknown. Previous studies have identified differences in the types and frequencies of gene mutations among endometrial cancers from Caucasians and African-Americans suggesting that the tumors from these two groups might have differing underlying genetic defects. We performed a gene expression microarray study in an effort to identify differentially expressed transcripts between African-American and Caucasian women’s endometrial cancers. Our gene expression screen identified a list of potential biomarkers that are differentially expressed between these two groups of cancers. Of these we identified a poorly characterized transcript with a region of homology to phospho serine phosphatase (*PSPH*) and designated phospho serine phosphatase like (*PSPHL*) as the most differentially over-expressed gene in cancers from African-Americans. We further clarified the nature of expressed transcripts. Northern blot analysis confirmed the message was limited to a transcript of under 1 kB. Sequence analysis of transcripts confirmed two alternate open reading frame (ORF) isoforms due to alternative splicing events. Splice specific primer sets confirmed both isoforms were differentially expressed in tissues from Caucasians and African-Americans. We further examined the expression in other tissues from women to include normal endometrium, normal and malignant ovary. In all cases *PSPHL* expression was more often present in tissues from African-Americans than Caucasians. Our data confirm the African-American based expression of the *PSPHL* transcript in endometrial cancer and also identify its expression in other tissues from African-Americans including ovary and ovarian cancer. *PSPHL* represents a candidate gene that might influence the observed racial disparity in endometrial and other cancers.

## Introduction

A racial disparity in incidence and survival exists for many human cancers. Understanding and addressing the reasons for these disparities is critical for reducing cancer burdens. A well described disparity in both incidence and survival outcome exists for endometrial cancer. Population based studies using data from both the National Cancer Data Base and Surveillance, Epidemiology, and End Results (SEER) have consistently shown that Caucasians (CA) are more likely to develop this cancer than are African-Americans (AA) even after controlling for hysterectomy (Hicks et al., 1998; Sherman and Devesa, 2003; Sherman et al., 2005). However, African-American women are about two times more likely to die from their disease than are Caucasians (Hicks et al., 1998; Ghafoor et al., 2002). The reasons underlying these disparities are complex and include social, cultural and biologic factors (Maxwell and Risinger, 2006). Several molecular genetic studies have identified differences in the prevalence of certain gene mutations or expression aberrations in cancers from AA and CA women. For example, the *TP53* tumor suppressor gene is mutated more frequently in endometrial cancers from AA women (Kohler et al., 1996). Similarly the HER2/Neu oncogene is more frequently up-regulated in endometrial cancer from AA women (Santin et al., 2005). In addition, a specific chromosome gain on chromosome one is more frequent in endometrial cancers from AA women (Morrison et al., 2010). Epigenetic methylation of the ribosomal DNA is also less prevalent in endometrial cancers from AA women (Powell et al., 2002). TP53, rDNA, and HER2 events are tied to more aggressive behavior and adverse outcome in endometrial cancer, suggesting they may be responsible in part for the outcomes disparity for AA women. In contrast, the *PTEN* tumor suppressor gene is more frequently mutated in cancers from CA and is associated with a more favorable outcome (Maxwell et al., 2000). It is important to note that African-Americans more often have non-endometrioid cancers and the above referenced gene changes are often more frequently associated with specific histotype e.g., TP53 and serous and PTEN with endometrioid type (Kohler et al., 1996; Maxwell et al., 2000; Wright et al., 2009).

Recently, we performed transcript expression studies in endometrial cancers (Risinger et al., 2003, 2005; Maxwell et al., 2005; Ferguson et al., 2006). These studies showed distinct expression related to histologic type as well as identifying transcripts associated with the microsatellite instability phenotype. In this study we specifically compared stage and grade matched endometrial cancers from AA and CA patients to identify whether distinct transcripts were associated with the race of the patient and, if so, whether these could serve as candidates for diagnosis, prognosis, or intervention. A previous study using a very similar study design found no global differences between AA and CA but did identify a small sub-set of genes differentially expressed. Although global differences between the two groups are not evident, we do identify a number of statistically differentially expressed transcripts, a sub-set of which were validated by quantitative real time PCR. Among those validated was that encoded by the gene for phosphoserine phosphatase like (PSPHL), which was found to be prominently expressed in endometrial cancer specimens from AA women and cloned an additional splice variant also preferentially expressed in AA women.

## Results

### Gene expression of african-american and caucasian endometrial cancers

**Figure 1:**
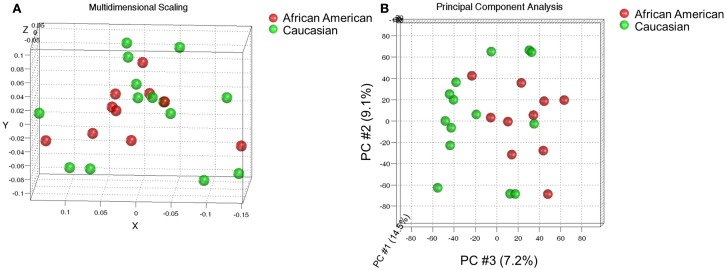
**Comparison of global expression profiles of endometrial cancers from African-American and Caucasian women**. **(A)** Multidimensional scaling using 1-correlation as distance metric did not distinguish the classes. **(B)** Principal component analysis indicates differential expression along PC #3 that explains 7.2% of the total variance may not be statistically significant. The expression data of about 18,500 transcripts detected in at least half of the cases were used for these analyses.

**Figure 2:**
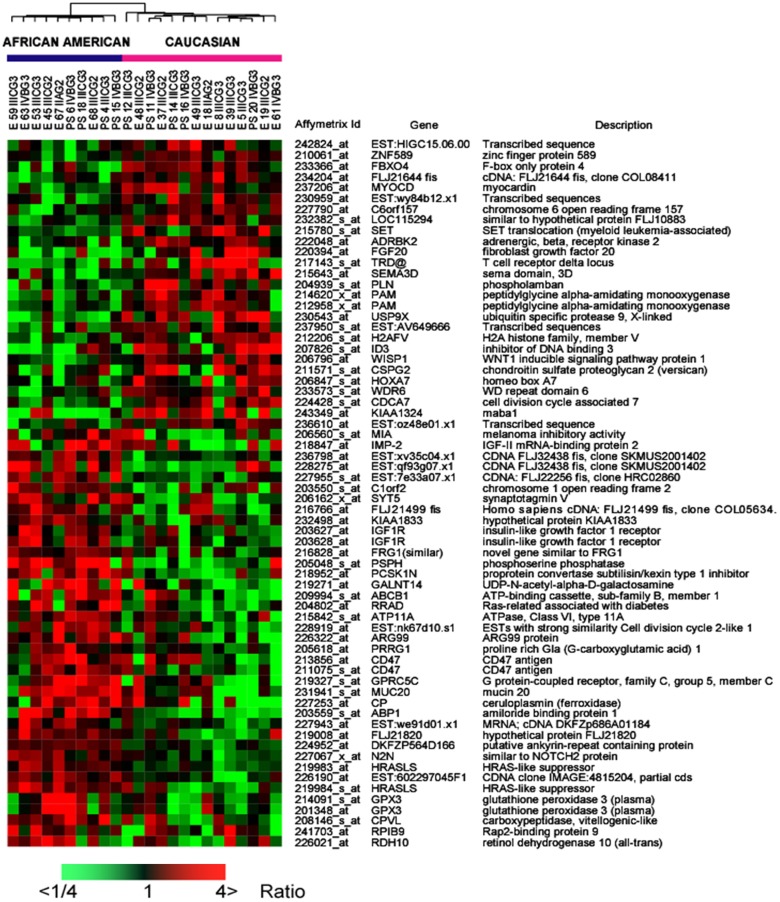
**Heat map of 66 transcripts differentially expressed between African-American and Caucasian by twofold and *p* < 0.005**. Expressions shown are mean centered. The red indicates increased and green down-regulated expression while black indicates mean value.

We performed a hybridization-based transcript expression analysis (Affymetrix) on a set of endometrial cancer specimens that were controlled for stage and grade and examined the global transcript expression of these cancers using multidimensional scaling. Cancers representing the groups were not found to be distinct indicating that race was not the chief determinant of gene expression between these two groups (Figure 1A). Class comparison tests between AA and CA indicated 341 transcripts at *p* < 0.005 with a global test *p*-value of 0.061. We further examined the data using principal component analysis (PCA). The first two principal components (PCs) explaining highest variance did not distinguish AA and CA classes. However, a sub-set of CA cases partially segregated from AA cases along PC #3 which explains only 7.2% of the total variance (Figure 1B). Despite the lack of global difference we determined which transcripts were differentially expressed between these groups by paired *t*-tests to control for histology, stage, and grade variations. We noted a total of 263 genes (325 transcripts) at *p* < 0.005 (Table S1 in Supplementary Material) – 66 of these marked by at least a twofold change are depicted in Figure 2.

### Quantitative validation of differentially expressed transcripts

**Figure 3:**
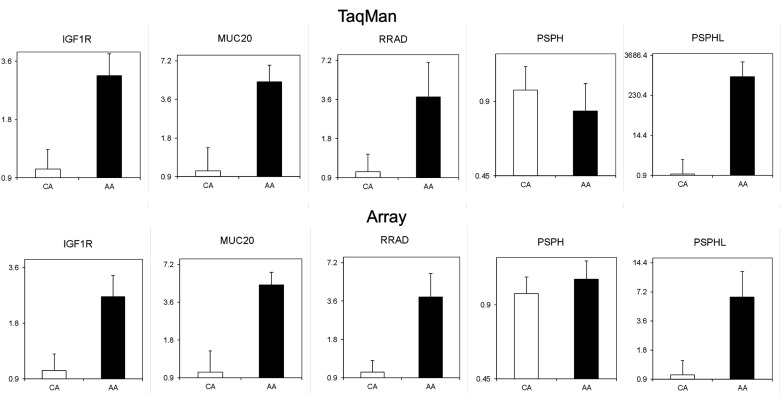
**TaqMan quantitative PCR validation of expression levels of IGF1R, MUC20, RRAD, PSPH, and PSPHL in endometrial cancers from Caucasian (*n* = 14) and African-American (*n* = 10) women is shown in top row**. The expressions were normalized to endogenous β-actin and then the ratios to Caucasian are shown. Bottom row is from array data of same samples.

Findings on gene expression microarrays require validation using alternate sample sets and more quantitative methodologies. We performed quantitative real time (qRT) PCR to validate the array findings for a sub-set of transcripts identified. Specifically we examined the Insulin-like Growth Factor Receptor 1, (*IGFR1*) Mucin 20, (*MUC20*), Ras Related Associated with Diabetes, (*RRAD*), and the Phosphoserine Phosphatase (*PSPH*) genes using qRT-PCR. Data from these assays performed on a panel of endometrial cancers from AA and CA confirmed the array findings for *IGF1R*, *MUC20*, and *RRAD*, but interestingly, qRT-PCR utilizing primers targeted to *PSPH* did not (Figure 3). Close inspection of the (Affymetrix) probe sequence indicated that the microarray probes were actually situated in a poorly described sequence locus on the opposite arm of chromosome 7. This transcript is alternatively designated as *CO9* or *PSPHL* due to sequence identity to parts of *PSPH*. This observation motivated the design of primers specific for *PSPHL* and re-analysis of the endometrial cancer specimens by qRT-PCR definitively demonstrates elevated expression of this transcript in AA as compared to CA patients (Figure 3).

### Overlap with previous published microarray data

**Figure 4:**
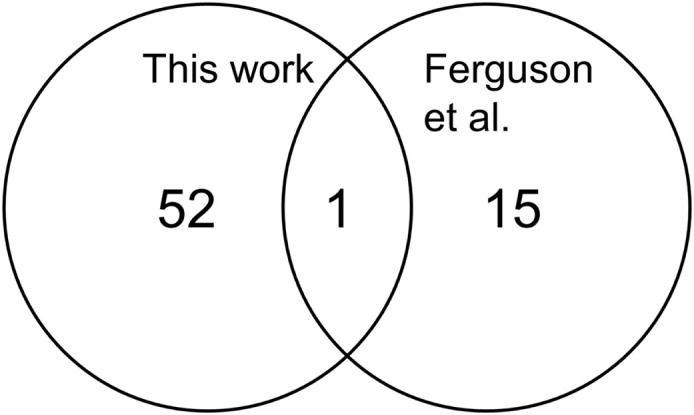
**Comparison of the numbers of differentially expressed transcripts from this work with the report of Ferguson et al. (2006)**. Only the transcripts altered by 1.4-fold at *p* < 0.001 between the endometrial cancers from African-American and Caucasian are shown. PSPHL is found to be highly expressed in African-American cases in both studies.

We compared the differentially expressed genes with those described by Ferguson et al. (2006). We noted that probe set mis-annotated for PSPH was the single overlapping gene when we compared those transcripts identified at *p* < 0.001 (Figure 4) This fact and the general observation that there were few genes identified and those with low *p*-values exhibit small fold differences for most genes further suggesting the effect of race on gene expression is not prominently delineated from this type of microarray analysis.

### Transcripts from the *PSPHL* gene

**Figure 5:**
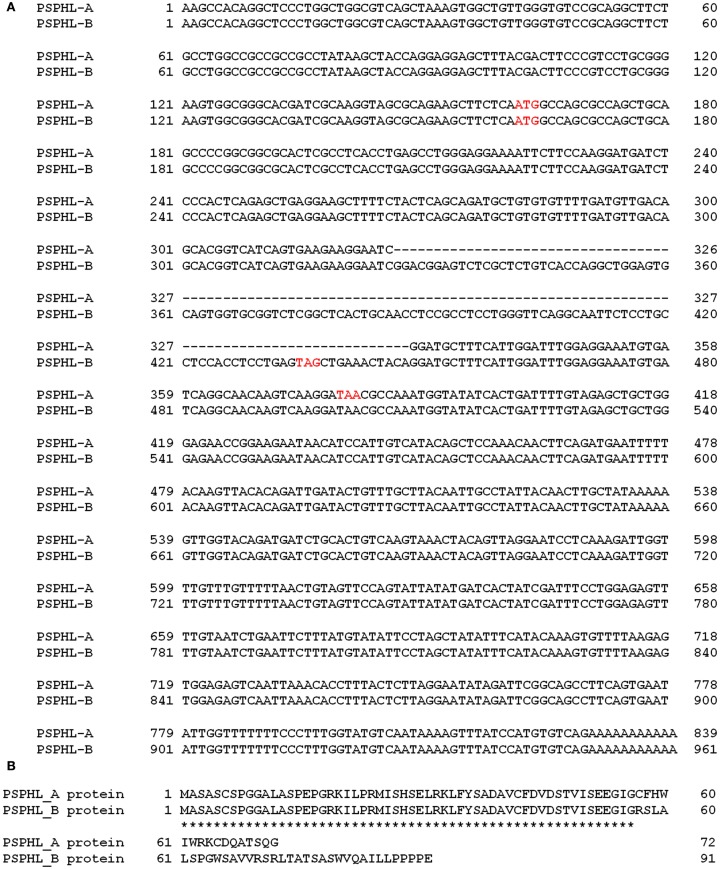
**(A)** mRNA homology of splicing variants of PSPHL PSPHL_A mRNA sequence (839 bp) is 100% match to the PSPH homolog C09 gene (503 bp) and l-3-phosphoserine-phosphatase homolog (839 bp; AJ001612). **(B)** Protein homology of splicing variants of PSPHL PSPHL_B has a novel spliced 122 bp sequence in the middle of PSPHL_A form alters the reading frame and utilizes a different stop codon.

**Figure 6:**
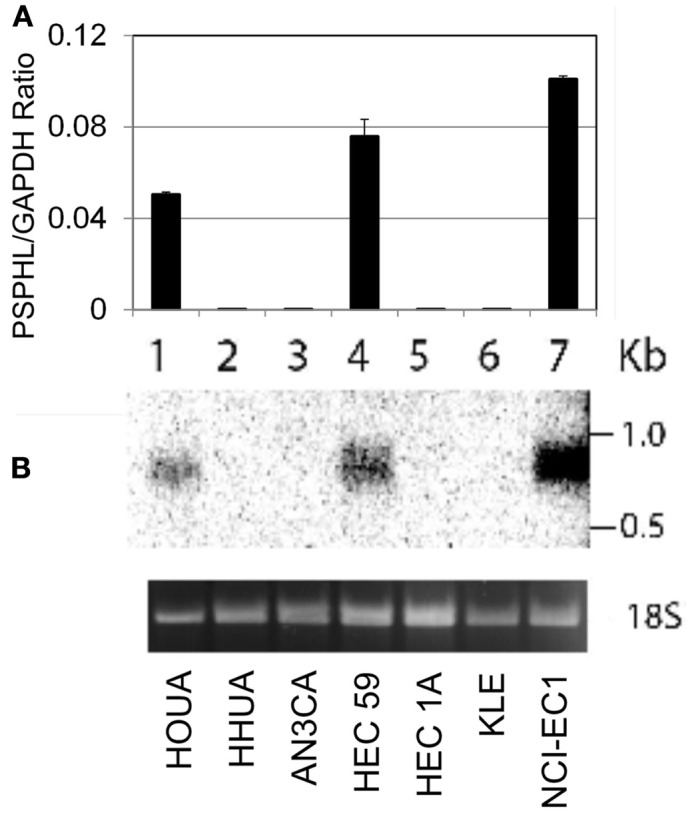
**(A)** Expression levels of PSPHL in uterus cancer cell lines using quantitative PCR. **(B)** Northern blot analysis using PSPHL specific probes, (1) HOUA (2) HHUA (3) AN3CA (4) HEC 59 (5) HEC 1A (6) KLE (7) NCI-EC1. Ethidium Bromide stain of the 18 s subunit for loading control.

Deposited transcript information for this locus is represented by one mRNA and three spliced ESTs (BG183407, BG196884, and BX380670). In addition a publication described a related *PSPH* sequence as the *CO9* gene and published a 840 bp long sequence with homology to exons of *PSPH* and matched the 3′ end of the consensus *PSPHL* transcript deposited in NCBI (Planitzer et al., 1998). To clarify the nature of the over-expressed sequence we amplified fetal cDNA using primers homologous to the predicted ORF of *PSPHL*. This PCR gave two prominent bands following agarose gel electrophoresis (data not shown). These PCR products were cloned in the pENTR Topo vector, resultant individual colonies isolated, and plasmid clones were sequenced. We confirmed the sequence of an isoform identical to the PSPHL ORF as well as a second isoform with an apparent alternative splice that matched no other deposited RNA. These sequences contain ORFs that predict small peptides with a similar n-termini with a PSPH homologous region but differing in their c-terminal end due to frame shifting of the ORF (Figure 5). Potential peptides generated from the two longest ORF from these isoforms are very small at 72 aa and 91 aa. We therefore examined whether the *PSPHL* transcript was incompletely cloned. We determined the expression of PSPHL in a panel of endometrial cancer cell lines using quantitative PCR and then probed RNA from some of these using a cDNA probe specific to *PSPHL* to determine the size of the *PSPHL* message. In cell lines expressing the transcript we noted a message that was less than 1 kb (Figure 6) suggesting that longer forms of PSPHL were not expressed in these endometrial cancers and missing from databases. We next performed 5′ and 3′ prime rapid amplification of cDNA ends (RACE) on a cDNA sample prepared from a pool of uterine carcinomas from African-Americans. Resultant clones were sequenced and compared to the deposited concensus PSPHL sequence in NCBI. There were two forms of *PSPHL* identified one of which matched to the consensus sequence. However the second matched the form we identified from ORF specific PCR and has not been described, which we designated form B. Our data indicate that two spliced forms of *PSPHL* were expressed in these cancers. The length of message on Northern analysis and cloned transcripts are consistent with two forms expressed in these tissues. A diagram of the *PSPHL* locus and predicted peptides is shown in Figure 5.

### *PSPHL* expression in normal and tumor tissues

**Figure 7:**
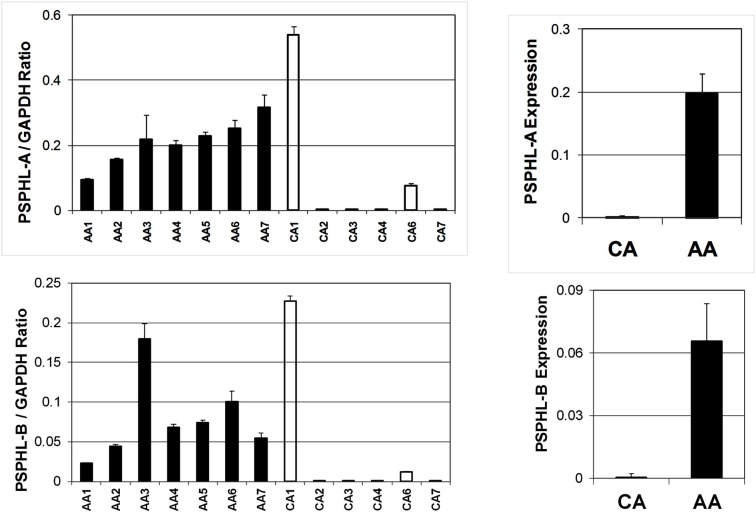
**Relative expression levels of PSPHL-A (top) and PSPHL-B (bottom) in normal endometrium for Caucasian and African-American women**. Left: individual cases; Right: averages. PSPHL-A: AA/CA ratio = 279, *p* = 0.007; PSPH:L-B AA/CA ratio = 146, *p* = 0.0065.

**Figure 8:**
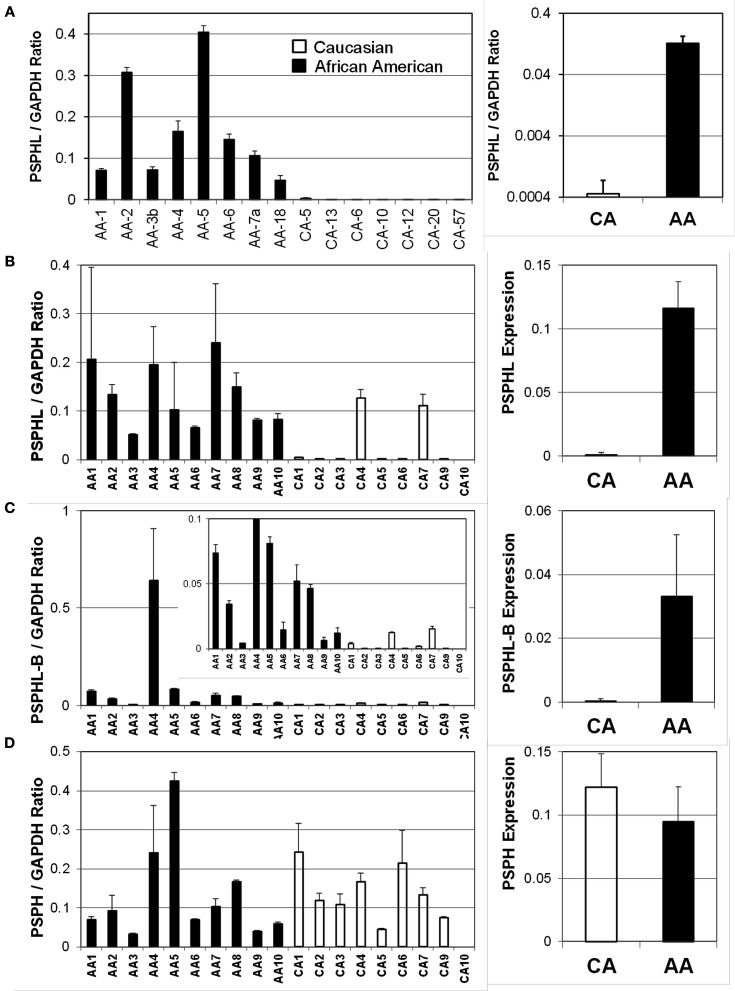
**TaqMan validation of expression levels for Caucasian (CA) and African-American (AA) women: (A) PSPHL-A in ovarian cancers, (B) PSPHL-A in normal ovary, (C) PSPHL-B isoform in normal ovary and (D) PSPH in normal ovary**. Left Individual cases; Right: geometric averages. Error bars indicate standard error. The inset of **(C)** shows ratios in expanded scale. AA/CA ratios: *A* = 233. *B* = 139. *C* = 92, *D* = 0.78; *p*-values *A* = 1.24 × 10^−7^; *B* = 0.00075; *C* = 0.001; *D* = 0.46.

**Figure 9:**
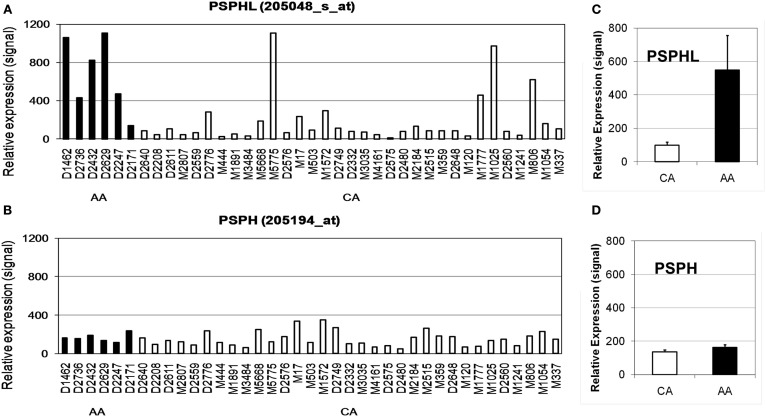
**Relative expression levels of (A) PSPHL and (B) PSPH in serous ovarian cancers from six African-American (AA) and 33 Caucasian (CA) women estimated using array data from Berchuck et al. (2009)**. **(C,D)** Geometric averages. PSPHL: AA/CA ratio = 5.56, *p* = 0.00046; PSPH: AA/CA ratio = 1.20, *p* = 0.39.

We further explored the expression of *PSPHL* in additional human tissue samples. We wished to determine if *PSPHL* was expressed in normal endometrium or if there was evidence that supported whether this transcript is differentially expressed in cancers from African-Americans. We examined *PSPHL* expression in a set of 13 tissue specimens of normal endometrium with seven being from AA and six from CA. Expression of *PSPHL* was more prominent in the AA specimens (Figure 7). We also found that *PSPHL* form B was expressed more frequently in AA tissues (Figure 7). To further examine *PSPHL* expression we examined normal and cancer tissues from the ovary. We examined a set of stage IIIc serous ovarian cancers and again noted a race difference (Figure 8). Similar to data from the endometrium, normal ovary tissue from AA displayed an elevated level of the transcript for *PSPHL* whereas there was no significant difference in levels of *PSPH* (Figure 8), with isoform B being elevated in AA (Figure 8). We next confirmed in a public array set of 39 serous ovarian cancers the expression of *PSPHL* preferentially in AA cancers compared to CA and no change in *PSPH* (Figure 9).

### *PSPHL* protein

**Figure 10:**
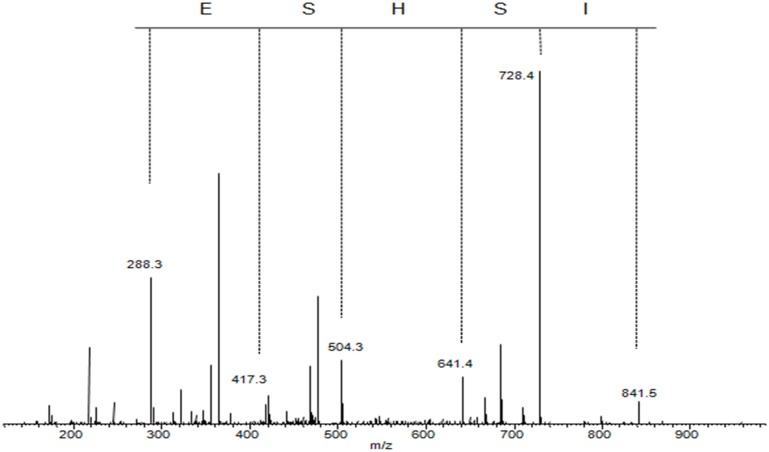
**Collision-induced dissociation spectrum of MISHELR, a unique peptide derived from trypsin digestion of PSPHL**.

**Figure 11:**
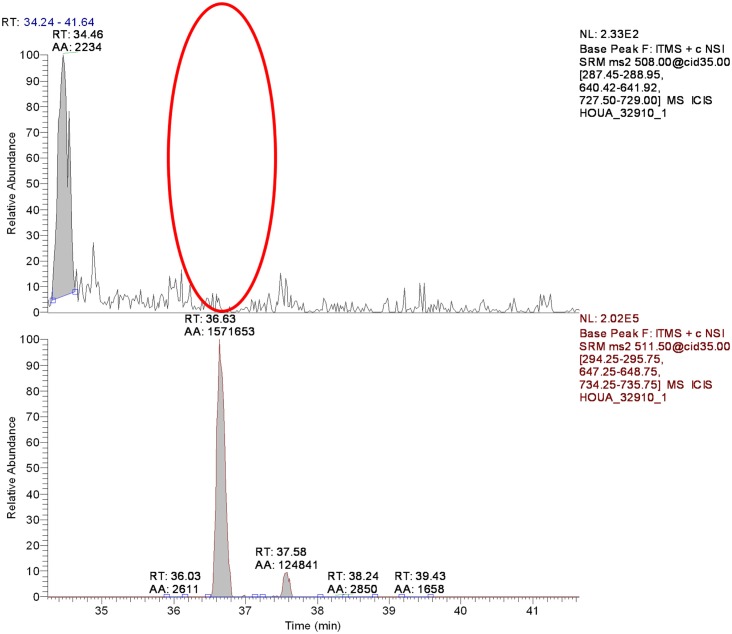
**Detection of the diagnostic MISHELR** **peptide of PSPHL in HOUA endometrial cancer cell lysate**. Top panel. Red oval indicates the area where the peptide should be detected, its absence indicating no PSPHL protein in this extract. Bottom shows the recombinant peptide when spiked in the extract.

Phosphoserine phosphatase (PSPH) is a 225 amino acid enzyme responsible for catalyzing the last step in the biosynthesis of serine from carbohydrates. The two transcripts correlating to putative phosphoserine phosphatase like protein (PSPHL) could encode a 72 or 91 amino acid homolog of PSPH. However, no evidence exists to date demonstrating a protein product from *PSPHL*. We cloned and expressed a tagged version of PSPHL in *E. coli* and a total lysate resolved by 1D PAGE and Coomassie staining revealed evidence of PSPHL at the predicted mass of 7.8 kDa (not shown). The prominent protein band was excised, in-gel digested with trypsin and peptides and analyzed by nanoflow reversed-phase liquid chromatography (nRPLC) coupled online with tandem mass spectrometry. The collision-induced dissociation spectra resulting from this analysis were searched using SEQUEST (ThermoFisher Scientific) against the human UNIPROT proteome database. The results confirmed the presence of a number of PSPHL peptides confirming the presence of the PSPHL gene product by mass spectrometry. Shown in Figure 10 is the CID spectrum from a unique peptide from PSPHL (MISHSELR), which unambiguously displays prominent fragment ions from the core sequence of the peptide. We utilized this methodology in attempts to detect the *PSPHL* gene product in human endometrial cancer cells. We examined cell lines with negative and positive endogenous PSPHL transcript expression. We did not identify any PSPHL peptides in either sample supporting the conclusion that the endogenous PSPHL is not found at the protein level (Figure 11). Furthermore it is notable that there exists no evidence in the literature that the *PSHPL* gene encodes for a functional protein product. Further, this gene has been annotated as likely being a pseudogene by the European Bioinformatics Institute^1^.

### Expression related to outcome in gynecologic cancer

**Figure 12:**
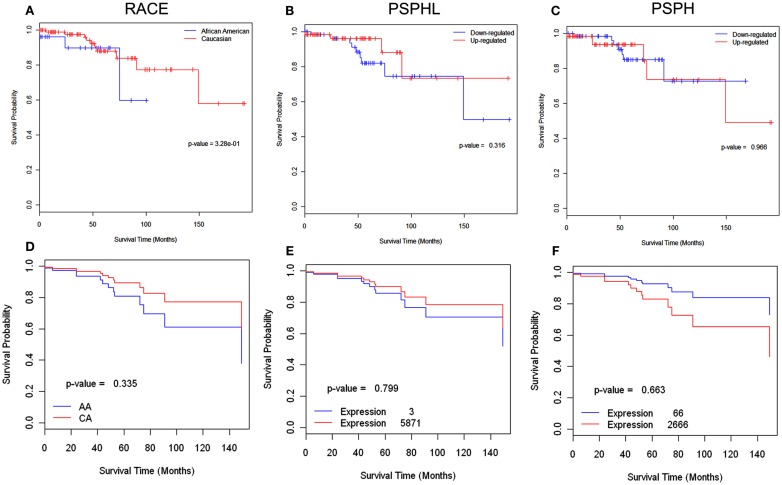
**Survival analysis of endometrial cancers using 131 cases (AA-26, CA-105) in which there were I3 (AA-3, CA-10) deaths in the study period of 16 years**. Top row Kaplan–Meier curves for **(A)** Race **(B)** PSPHL, and **(C)** PSPH. Up and down-regulations are from median level. The *p*-values are not statistically significant due to small number of events available for the analysis. **(D–F)** Cox regression analysis of same data showing estimated survival curves: **(D)** for AA and CA, **(E)** minimum and maximum signal levels of PSPHL and **(F)** minimum and maximum signal levels of PSPH.

**Figure 13:**
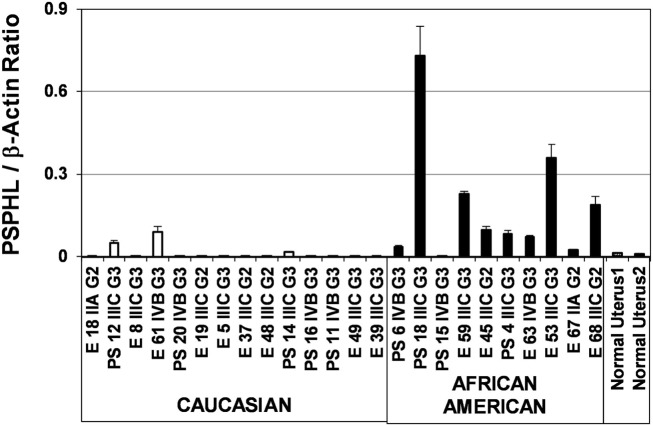
**Expression of PSPHL in endometrial cancers from Caucasian**. African-American women and normal uterus.

**Figure 14:**
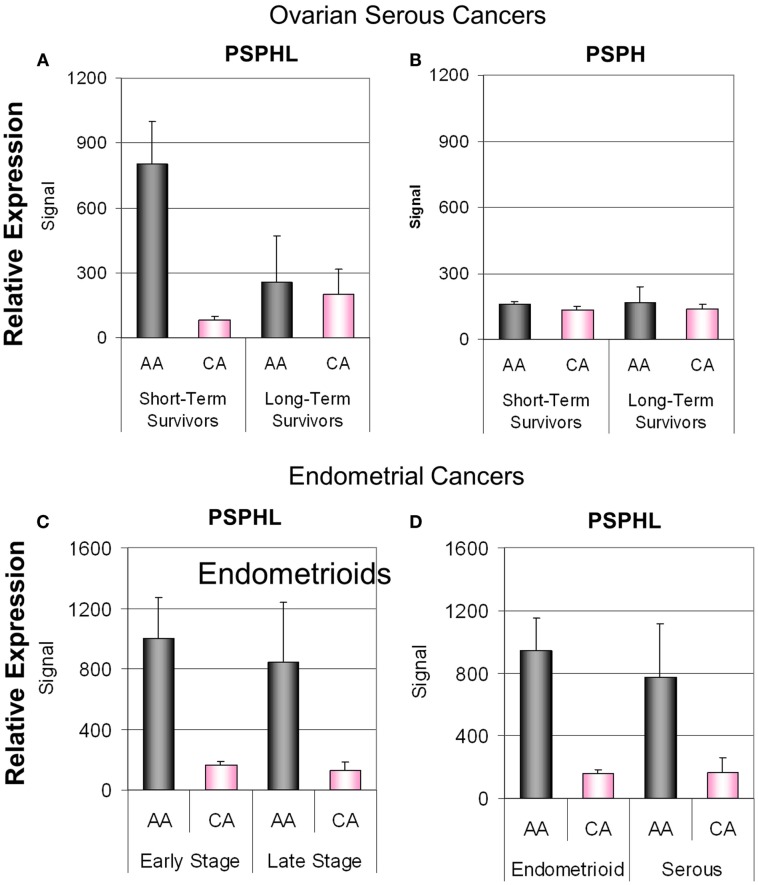
**(A)** Differential expression of PSPHL in ovarian cancers of short-term survivors. **(B)** Relative expressions of PSPH in the same ovarian cancers (data from Berchuck et al., 2009). Differential expression of PSPH in endometrial cancers: **(C)** by stage and **(D)** by histologic type. AA, African-American; CA, Caucasian; Signal, geometric average signal from Affymetrix micro arrays.

African-Americans exhibit significant health disparities compared to Caucasians in the United States. We examined two array data sets of endometrial and ovarian cancer to determine whether there were differences in expression of PSPHL related to outcome. First we examined a set of 131 uterine cancers for which we knew race and outcome and that had been previously arrayed on Affymetrix chips. These data include 26 AA cases with 3 deaths and 105 CA cases with 10 deaths in the study period of about 16 years between 1989 and 2007. However, we noted few deaths overall in this group of cancers and even fewer among African-Americans. The small number of events limited the strength of survival analysis. We examined the effect of race in this dataset and noted that Caucasians actually survived better (Figures 12A,D) though the result is not statistically significant. We confirmed higher expression of PSPHL in African-Americans cancers in this data set regardless of histologic type (Figures 13 and 14). However, when we examined the overall association of PSPHL expression in this dataset we found there was no difference in survival between high expression and low expression of PSPHL or PSPH (Figures 12B,C).

We next examined a data set of short- and long-term survivors of advanced serous carcinoma of the ovary. We confirmed the higher expression of PSPHL in African-Americans’ cancers but again noted an insignificant association with outcome (Figures 12E,F). We did note a high level of PSPHL expression in some short-term survivors but the number of cases was small and when the data are entered in a continuous model of survival statistically insignificant (Figure 14).

## Discussion

A well recognized racial disparity in incidence and survival outcome exists for endometrial cancer patients in the United States. The racial disparity in outcome is likely multi-factorial. Some reasons include access and utilization of healthcare that may be due to unequal treatment and mistrust of the medical community, as well as differences in cultural acceptance of disease and its fate. However, some authors have suggested that the disparity cannot be explained in total by non-biological factors (Sherman and Devesa, 2003). African-American women tend to be diagnosed with more advanced disease and with adverse histologic types and higher grade, than do Caucasians. These factors are reflective of a more aggressive type of cancer (Steinhorn et al., 1986). Although the endometrial cancers diagnosed in African-American women are often non-endometrioid and poorly differentiated, survival remains worse for African-Americans in investigations that control for stage, grade, histology, and surgical treatment (Randall and Armstrong, 2003).

Molecular genetic data indicates differences in the kinds of mutations that occur in tumors from each group. Several studies have examined the mutational frequency of genes commonly mutated and involved in the pathogenesis of endometrial cancer (Risinger et al., 1997; Tashiro et al., 1997; Suzuki et al., 1998; Stambolic et al., 2000). In addition to differences in DNA mutations several studies have examined other changes to include microsatellite instability, HER2/NEU over expression, and rDNA methylation (Maxwell et al., 2000; Powell et al., 2002; Santin et al., 2005). Based in part on these observations Ferguson et al. (2006) examined the gene expression from African-American and Caucasian women and found few statistically differentially expressed genes. We performed a similar type study which is described in this manuscript and found a few more statistically significant genes but largely confirm that underlying expression due to the patients race was not apparent in global expression. We did identify a set of genes that were statistically significant and might represent candidates for evaluation on racial disparity of this disease. However we chose to focus most of our validation efforts on the single probe set that was significantly changed in both sets of data and represented not only robust statistical significance but a significant fold change in expression as well. Interestingly the probe set targeted a poorly described transcript with homology to PSPH. PSPH, Phosphoserine phosphatase (PSP) normally functions in the conversion of l-phosphoserine to l-serine. Serine is one of the amino acids necessary for protein and nucleotide synthesis. Recently several reports implicate PSPH as a stem cell marker (Skalnikova et al., 2008). Furthermore PSPH has been identified as a gene up-regulated in gastric micrometastasis (Shimomura et al., 2004). Recently PSPH has been suggested to also be involved in cell to cell signaling. However we noted an inability to validate our array finding using a PSPH specific TaqMan assay. We explored the reasons for this and noted that the Affymetrix probes were more likely targeting the poorly described C09 or PSPHL gene also located on chromosome 7. We were able to confirm the original array findings when we used a probe set specific to PSPHL and not PSPH.

We noted no functional data in the literature for PSPHL and were uncertain to the completeness of the annotation. No protein has been identified for PSPHL and its function as a non-coding RNA or as a protein is unknown. Although it contains a region of exact homology with PSPH the predicted peptide from the described gene would encode a very small protein. We further characterized the PSPHL transcript by performing Northern analysis and found that PSPHL specific probe targeted a roughly 900 bp message suggesting that there were not larger PSPHL transcripts. When we performed PCR on the predicted ORF of PSPHL we noted two distinct bands. We cloned these and found an alternative isoform of PSPHL that adds an additional exon and at that point also changes the open reading frame. We performed 5′ and 3′ RACE and were able to isolate PCR clones representing only these two splice forms of *PSPHL* that were predicted to produce 72 or 91 kDa peptides.

Recently Wei et al. (2011) examined the gene expression from endothelial cells from African-Americans and Caucasians also in an effort to identify underlying gene expression differences related to health disparity. They too noted increased PSPHL expression in endothelial cells in African-Americans and the mis-annotated PSPH probe set. Furthermore they examined the effect of PSPHL expression on its enzymatic l-serine phosphatase activity and found the PSPHL was enzymatically inactive. Additional studies by them indicated that PSPHL did not interfere with normal PSPH activity.

Many cancer sites exhibit racial disparities in outcome and incidence. Prostate and breast cancers cancer disproportionaltely affects AA. Similar expression array studies have been performed for prostate and breast cancers. Wallace et al., identified *PSPHL* as one of the most racially differentially expressed genes in prostate cancers. Expression was higher in AA men and along with *CRYBB2* was an accurate two gene classifier for AA status in prostate cancer tissue but not corresponding normals (Wallace et al., 2008). Similarly this same group identified *PSPHL* over expression in AA women compared to CA women’s breast cancers and surrounding stroma (Martin et al., 2009). Again *PSPHL* and *CRYBB2* were demonstrated to be an accurate two gene classifier for AA status in tumors. PSPHL was one of five genes necessary to classify the stroma of these patients.

Importantly we examined whether PSPHL expression was a tumor derived characteristic or whether the expression was present prior to neoplastic transforming processes. In two different tissue types uterus and ovary *PSPHL* was expressed more often in both normal and malignant tissue from African-Americans. Indicating that PSPHL expression is not solely a tumor derived characteristic. This is also supported by the findings in breast, prostate, and endothelial cells (Wallace et al., 2008; Martin et al., 2009; Wei et al., 2011). It also suggests that if *PSPHL* plays any role in the racial disparity of endometrial cancer it could affect numerous processes. Raising the questions of whether presence of PSPHL actually serve to protect endometrial transformation in African-Americans where the disease is less prevalent? Or does it affect some biologic function that could adversely affect outcome such as drug response, invasion, metastatic spread? Or is *PSPHL* expression just reflective of an underlying racial difference in gene expression and have no relation to endometrial cancer. In this regard we like others could not find evidence for a PSPHL peptide in human cells despite using sophisticated proteomic methods on cells highly expressing the PSPHL transcript. It is likely if PSPHL has function in cells it is occurring through an RNA mediated mechanism. Recently functional roles for LncRNAs have come to light (Prensner and Chinnaiyan, 2011). The abundance of mRNAs may also regulate other genes by a ceRNA modulation of miRNA levels at their 3′ UTRs (Poliseno et al., 2010; Salmena et al., 2011; Tay et al., 2011). It is clear that future studies are required to assess the biologic function if any of this transcript.

We did examine two gene expression array datasets with clinical survival endpoints and designated tissues by race to gain insight on whether *PSPHL* expression is related to survival. We confirmed the preferential expression of *PSPHL* in African-Americans as compared to Caucasians cancers in both sets one endometrial and one ovarian. As we found throughout our studies a significant number of cancers from Caucasian do express *PSPHL*. This fact allows us to track survival related to the genes expression rather than using its expression as a racial surrogate. In these analyses we had access to a dataset of 133 endometrial cancers of which only 13 expired. We did not see any association with survival in this set perhaps reflecting the overall good survival in this data set. It will be important to analyze the relevance of PSPHL in larger datasets with more African-Americans and a greater number of adverse survival events. Similarly we noted a non-significant association with survival in serous ovarian cancers a tumor type generally not associated with a racial disparity in outcome. Interestingly we noted that among the short-term survivors in a large set of advanced ovarian cancer that PSPHL was highly expressed in AA tumors although this data was generated from only a few individuals.

In summary we identified that the *PSPHL* transcript is differentially expressed in endometrial cancers from African-Americans and Caucasians. This tumor type occurs more frequently in Caucasians but is more deadly in African-Americans. PSPHL was also preferentially expressed in normal endometrium of African-Americans as well as normal and malignant ovary tissues. *PSPHL* may ultimately be found to serve as a prognostic marker for these diseases and could function in numerous cellular processes to include either tumor suppression or by affecting biologic and other treatment events related to outcome.

## Materials and Methods

### *PSPHL* transcript characterization

Transcript size was determined by Northern blot of endometrial cancer cell line total RNAs isolated from tumor biopsies. Transcript specific ^32^P labeled probes were generated by random priming from inserts excised from sequence verified vector containing the ORF of PSPHL. Hybridized blots were washed at high stringency and exposed to phosphor screens. Phosphor screens were scanned using a Molecular Dynamics STORM (GE Healthcare, UK) phosphorimaging system for quantitative measurements. PCR primers designed to suspected PSPHL ORF were ATGGCCAGCGCCAGCTGC and TTATCCTTGACTTGTTGCCTGATC. Rapid Amplification of cDNA Ends (SMART RACE) was performed according to the manufacturers recommendations and using a nested primer strategy. 5′ and 3′ cDNAs were prepared from a pool of four uterine carcinomas from African-American women. Primers utilized in 5′ RACE were CCAGCAGCTCTACAAAATCAGTG and ATTCTTCCGGTTCTCCCAGCAGC. Primers used for 3′ RACE were ATGGCCAGCGCCAGCTGC (same as ORF above) and AAGCTTCTCAATGGCCAGC. PCR products were cloned in pENTR TOPO vectors (Invitrogen) according to manufacturers recommendations. Isolated plasmid DNAs were sequenced with vector based primers M13-F and M13-R.

### Cell lines

AN3CA, KLE, HEC-1-A were obtained from the American Tissue Type Collection, Rockville Md. HEC-59, HOUA, and HHUA were a kind gift from Hiroyuki Takahashi, Jikei University, Tokyo Japan. NCI-EC1 was developed by the corresponding author at the National Cancer Institute (NCI) from a Stage IIIC endometrioid cancer. Cells were grown in DMEM/F-12 supplemented with 10% fetal bovine serum and maintained in a humidified 5% CO2 incubator.

### Tissue specimens

Flash frozen cancer specimens were obtained from patients undergoing surgery for uterine cancer at Duke University Medical Center and were part of a microarray data set previously reported (Maxwell et al., 2005). All tissues were collected under an IRB approved protocol at Duke University Medical Center. Specimens were harvested within 30 min of specimen removal at the time of surgery. Each uterine tumor was then frozen until the time of the analysis. The set of endometrial cancers selected for this analysis included 10 African-Americans and 14 Caucasians. Race determination of patients reflected self-described racial status. Only specimens from African-Americans for which there were specimens from Caucasians that were matched by stage, grade, and histology were considered for analysis. Tissue specimens were evaluated by H&E to confirm that all specimens comprising the matched pairs contained at least 50% or greater cancer cells. During preparation of the specimens for analysis, care was taken to macroscopically dissect the cancer away from any adjacent myometrium. Endometrial, ovarian, and breast samples used for quantitative PCR validation of array data were obtained from the cooperative tissue network, Southeast region. Tumor RNAs used in PSPHL cDNA cloning were obtained from Memorial Health University Medical center under an approved IRB protocol. Tissue samples were subjected to RNA isolation following laser capture of epithelial cell fraction using TRIzol followed by an additional level of purification with the Rneasy Kit (Qiagen, Valencia, CA, USA). The integrity of each of the RNA samples was confirmed using denaturing gel electrophoresis.

### Gene expression analysis

The gene expressions were assessed using the Affymetrix human genome HG-U133A and HG-U133B Genechips. Approximately 5 μg of total RNA from each sample was labeled using the high yield transcript labeling kit (ENZO) and labeled RNAs were hybridized, washed, and scanned according to the manufacturers specifications (Affymetrix Inc., Santa Clara, CA, USA). Affymetrix Microarray Suite 5.0 software (MAS5) was used to estimate transcript signal levels from scanned images by one step Tukey’s biweight algorithm. The signals on each array were normalized to a trimmed mean value of 500 excluding the lowest 2% and highest 2% of the signals. An Affymetrix probe set representing a unique Gene Bank sequence is referred as a transcript hereafter for convenience. Unsupervised data analysis was performed using Multidimensional scaling where the distance metric was 1-ρ (ρ is correlation coefficient) and Principal Component Analysis (PCA) by minimizing the correlations (using R-Statistical package). About 18,500 transcripts having detection *p*-value <0.065 in at least half of the arrays were included in these analyses. Binary class comparison was performed on the different racial groups using BRB Array tools software (BRB Array tools ver. 3.0c, Richard Simon, Amy Peng, Biometric research branch, NCI, NIH^2^). The transcripts having detection *p*-value >0.065 in more than 95% of the arrays were eliminated for statistical comparisons of classes. Class comparison using two-sample *t*-tests between AA and CA indicated 341 differentially expressed transcripts at *p* < 0.005. The significance of finding these 341 transcripts was estimated by a global test using random permutations of class labels (BRB Array Tools) which gave a *p*-value of 0.061. This lack of global differential expression likely arises from variations in clinical factors. In order to account for variations in histology, stage and grade, we considered matched-pair analysis between AA and CA cases. The matching of 10 AA cases with 14 CA cases resulted in 14 pairs in which four AA cases were duplicated. Class comparison of these matched pairs by paired *t*-tests indicated 325 transcripts at *p* < 0.005 with a global test *p*-value of 0.045. There were 66 transcripts altered by twofold among these. Hierarchical clustering of these transcripts was performed on mean centered gene expressions using 1-ρ as distance metric and average linkage algorithm (Salmena et al., 2011). The heat map was color-coded using red for high expression and green for low expression compared to the array average in black. All the statistical calculations were performed on the logarithmic values of signals to the base 2. Survival analysis was done using “survival” package contributed to R-project^3^. A uterine cancer data set including 26 AA cases (with three deaths) and 105 CA cases (with 10 deaths) in a study period of about 16 years between 1989 and 2007 was used for Kaplan-Meier plots and for Cox regression analysis. Log-rank and Wald tests were used to calculate *p*-values of KM and Cox regression analyses respectively. Log-rank test of AA vs. CA cases indicated a *p*-value of 0.33. Cox proportional hazards regression analysis indicated poor survival of AA cases at Wald test *p*-value of 0.34. There was no significant difference in KM curves of the cases having PSPHL expression below the median and above the median level.

### Validation of gene expression using quantitative PCR

The expression of genes chosen for validation were determined by mulitplex PCR using TaqMan^®^ Gene Expression Assays purchased from Applied Biosystems, (Foster City, CA, USA) with endogenous β-actin as reference. Samples were run on the ABI Prism^®^ 7700 Sequence Detection System according to manufacturer’s suggested protocols. The relative quantitation was done using the comparative *C*_T_ threshold cycle (*C*_T_) method, and mean of triplicate *C*_T_ measurements was calculated for each sample and the ratios to β-actin were determined. The geometric average of the mean ratios for each racial group was presented with the standard error of mean values as error bars.

### Liquid chromatography-tandem mass spectrometry

The prominent gel band at 8 kDa was excised and in-gel digested with trypsin as previously described (Shevchenko et al., 2006). Peptide digests were analyzed in triplicate by reversed-phase liquid chromatography (Ultimate 3000, Dionex Corporation, Sunnyvale, CA, USA) coupled online to a linear ion trap MS (LTQ-XL, ThermoFisher Scientific, San Jose, CA, USA) as previously described (Hood et al., 2010). Tandem mass spectra were searched against the UniProt human protein database (10/08 release) from the European Bioinformatics Institute^4^, using SEQUEST (ThermoFisher Scientific). Additionally, peptides were searched for methionine oxidation with a mass addition of 15.9949 Da. Peptides were considered legitimately identified if they met specific charge state and proteolytic cleavage-dependent cross correlation scores of 1.9 for [M + H]^1+^, 2.2 for [M + 2H]^2+^, and 3.5 for [M + 3H]^3+^, and a minimum delta correlation of 0.08.

## Supplementary Material

The Supplementary Material for this article can be found online at http://www.frontiersin.org/Women’s_Cancer/10.3389/fonc.2012.00065/abstract

## Conflict of Interest Statement

The authors declare that the research was conducted in the absence of any commercial or financial relationships that could be construed as a potential conflict of interest.

## Footnotes

^1^http://www.uniprot.org/uniprot/O15172.

^2^http://linus.nci.nih.gov/BRB-ArrayTools.html

^3^http://www.r-project.org/index.html

^4^http://www.ebi.ac.uk/integr8
